# Case Report: Severe Eosinophilic Asthma Associated With ANCA-Negative EGPA in a Young Adult Successfully Treated With Benralizumab

**DOI:** 10.3389/fphar.2022.858344

**Published:** 2022-04-07

**Authors:** Luisa Ricciardi, Daniel Griscti Soler, Alessandra Bennici, Silvia Brunetto, Giovanni Pioggia, Sebastiano Gangemi

**Affiliations:** ^1^ Department of Clinical and Experimental Medicine, School and Operative Unit of Allergy and Clinical Immunology, Policlinico “G. Martino”, University of Messina, Messina, Italy; ^2^ Institute for Biomedical Reasearch and Innovation, National Research Council of Italy (IRIB-CNR), Messina, Italy

**Keywords:** benralizumab, asthma, EGPA, young adult, eosinophils

## Abstract

**Background:** Traditionally, Eosinophilic Granulomatosis with Polyangiitis (EGPA) has been treated with systemic corticosteroids and immunosuppressants. In recent years, therapeutic efforts have been directed towards targeting eosinophils which represent a major player in the pathogenesis of EGPA. In 2017 the Food and Drug Administration (FDA) approved mepolizumab, a humanized monoclonal antibody targeting interleukin 5 (IL-5) which reduces the production and survival of eosinophils, already used to treat severe eosinophilic asthma, for the management of EGPA. Benralizumab is a humanized monoclonal antibody that targets the IL-5 receptor and is indicated in the treatment of severe eosinophilic asthma.

**Case description:** We describe the case of a young female with a positive history of severe eosinophilic asthma associated with EGPA, treated successfully with benralizumab.

## Introduction

Eosinophilic granulomatosis with polyangiitis (EGPA) is a systemic vasculitis which affects small to medium vessels (Fagni et al., 2021) During the 2012 Chapel Hill Consensus Conference regarding the nomenclature of systemic vasculitis, the term “Churg-Strauss” was replaced by “EGPA” (Jennette et al., 2013) In the pathogenesis of EGPA, eosinophils play an important role, hence they are a therapeutic target (Khoury et al., 2014). In fact, mepolizumab, a monoclonal antibody targeting IL-5, an important cytokine in the differentiation and maturation of eosinophils, was authorized by the FDA for the treatment of EGPA in 2017 after demonstrating safety and efficacy (Wechsler et al., 2017). Benralizumab is a humanized monoclonal antibody that targets the IL-5 receptor and is indicated in the treatment of severe eosinophilic asthma. We describe the case of a young patient with severe eosinophilic asthma associated with EGPA who has been treated with benralizumab since March 2021 and has since managed to stop oral corticosteroids (OCS) completely.

## Case Presentation

**FIGURE 1:**
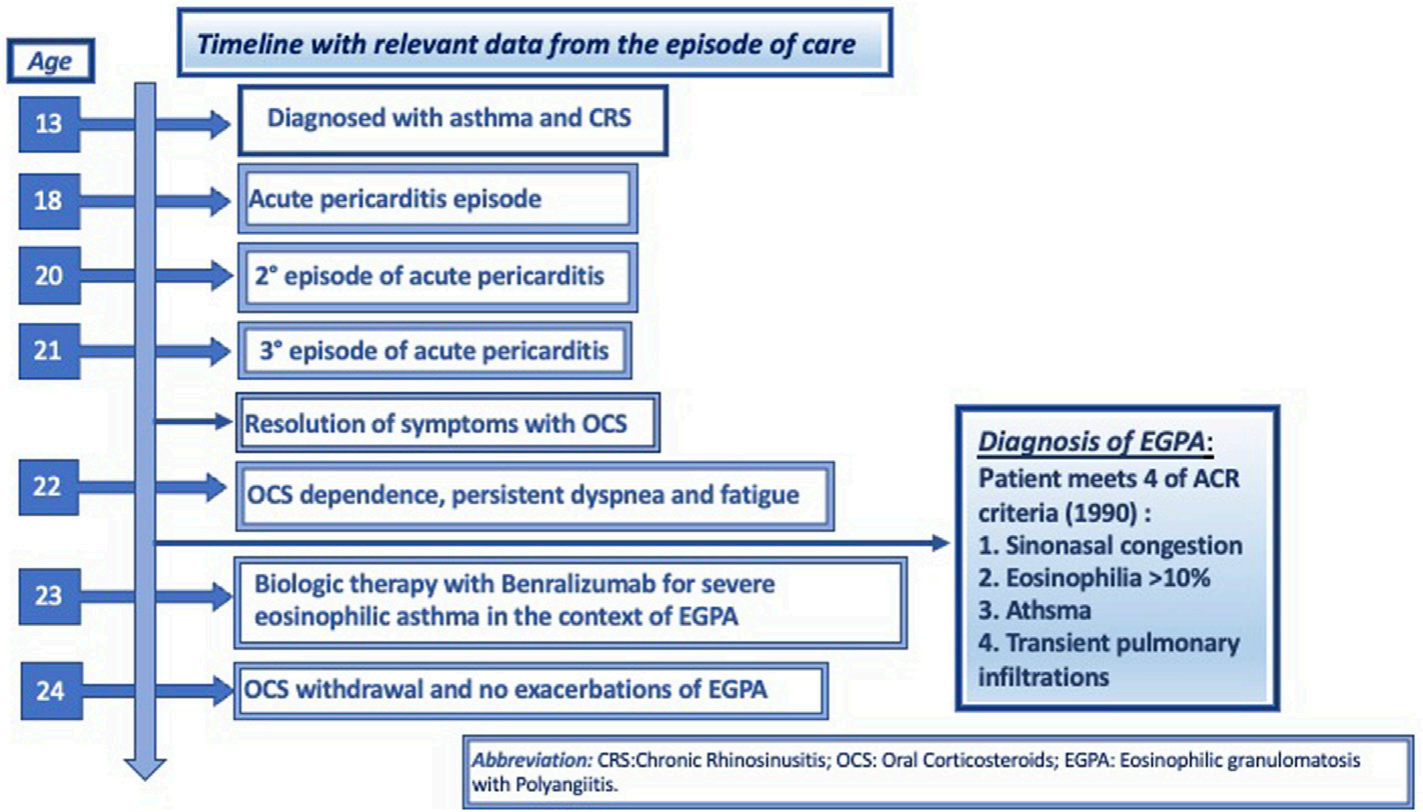
Timeline with relevant data from the episode of care, according to CARE case report guidelines.

**FIGURE 2:**
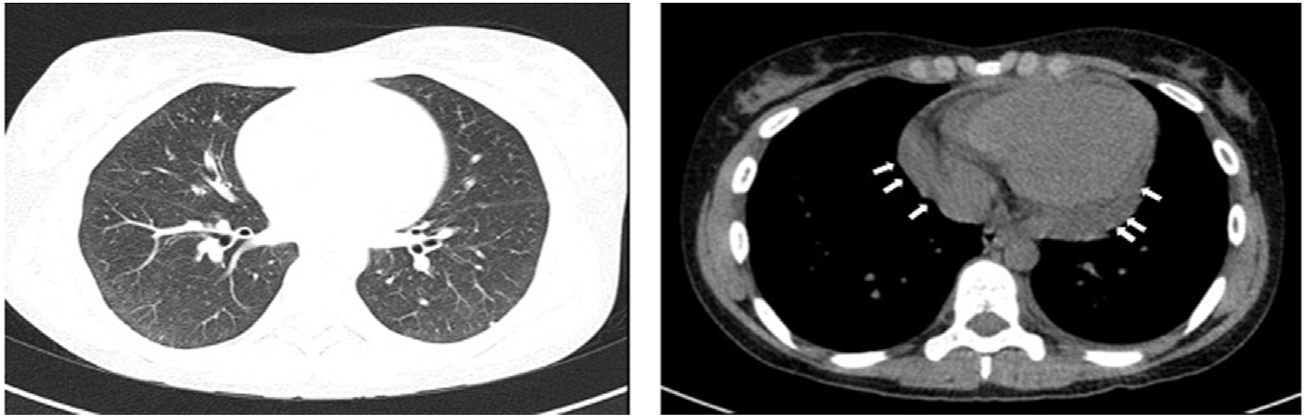
Thorax CT scan shows interstitial lung thickening with peripheral opacities. Thorax CT scan shows small pericardial effusion, as indicated by white arrows.

**FIGURE 3:**
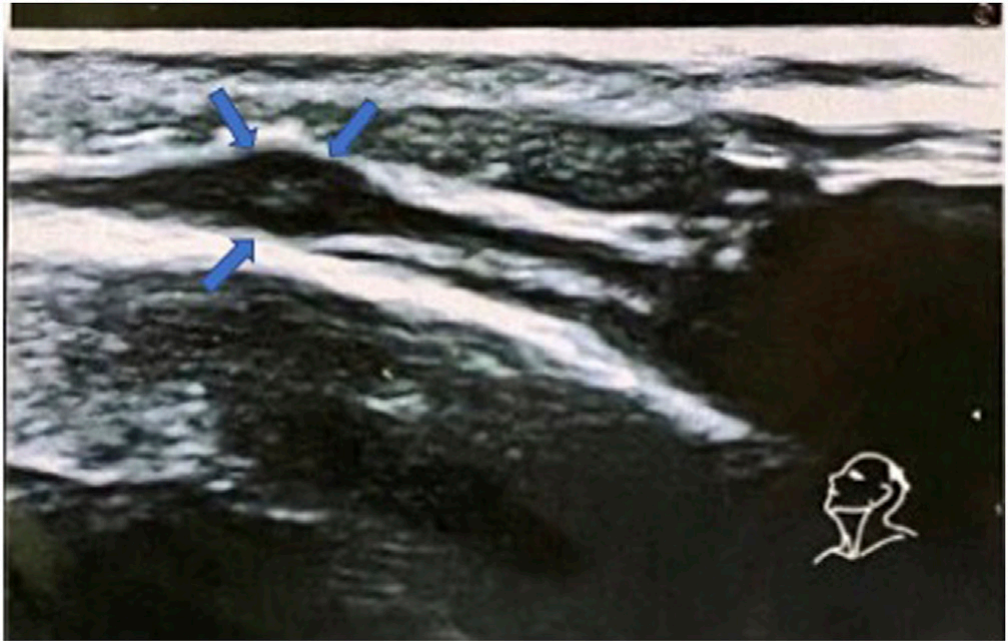
Ultrasound images of scalp nodules showing fibrotic and granulomatous characteristics.

We report the case of a 22-year-old female with a history of chronic rhinosinusitis, severe eosinophilic asthma, purpura, subcutaneous nodules on her scalp and recurrent episodes of pericarditis, who presented with worsening asthma, fatigue, and malaise despite being treated with prednisone 7.5 mg daily. As demonstrated in Figure 1, she had been diagnosed with asthma when she was thirteen years-old, for which she used ICS/LABA combinations. She used hypertonic saline nasal spray as needed to relieve her nasal symptoms. At the age of 18, she was hospitalized with acute pericarditis; on admission, blood tests showed an absolute eosinophilic count (AEC) of 3,319/mm^3^. A chest CT showed bilateral pulmonary peripheral opacities and a small pericardial effusion (as shown in Figure 2). She was treated with oral prednisone on a tapering schedule for 3 weeks with improvement of symptoms and a return to normal of her blood eosinophils. Two years later, she presented with a second episode of acute pericarditis and was treated with a tapering course of oral corticosteroids (OCS) without need for hospitalization. Later that same year she developed a nodular rash on her scalp with concomitant painful cervical lymphadenopathy, fever, and joint pains. An ultrasound of the scalp nodules in the parietal region reported thickening of the epicranial aponeurosis without vascular signals, compatible with fibrotic-granulomatous lesions, as seen in Figure 3. They resolved after a short course of OCS. The following year she was hospitalized again after developing pericarditis a third time. A cardiac MRI showed signs of myocarditis, but this was latent as there was no change in ejection fraction observed on transthoracic echocardiogram and the patient had no concomitant clinical features of heart failure. At hospital she was treated with colchicine and a few days later developed a palpable purpuric rash on her lower limbs. The latter was not biopsied. Due to recurrent pericarditis episodes and an AEC of 3,683/mm^3^, she was commenced on long-term prednisone starting at 50 mg daily tapering to 5 mg daily over 4 months, with complete clinical resolution of her rhinitis, asthma, recurrent pericarditis episodes and cutaneous rashes. On a stable dose of 5 mg daily and feeling well, she decided to suspend treatment on her own accord 6 months since starting prednisone. However off corticosteroid treatment, she began to report general malaise and dyspnea. Two months off prednisone, she developed a new papulonodular rash on her scalp with painful cervical lymphadenopathy and low-grade fever, thence, OCS was restarted at 7.5 mg daily. After a few months she still reported persistent dyspnea and fatigue, for which she decided to attend the Allergy and Clinical Immunology department at our hospital.

Autoimmune screening for our patient had been carried out previously with ANA titer levels ranged between 1:160 and 1:320 but had normalized on recent blood tests. ENA, ANCA and rheumatoid factor were consistently negative. Anti-phospholipid IgM levels were mildly elevated. Serum protein electrophoresis showed hypergammaglobulinemia but subsequent serum and urinary immunofixation testing were normal. Based on the patient’s history, a diagnosis of ANCA negative EGPA was given. Our patient met four of the ACR criteria established for EGPA: asthma, chronic rhinosinusitis, eosinophilia >10% and transient pulmonary opacities. Her clinical history included granulomatous scalp nodules and purpura suggestive of EGPA, even if biopsy of these lesions was not performed. Birmingham Vasculitis Activity Score (BVAS) were five points for persistent score and six points for new/worse score.

Despite treatment with 7.5 mg of prednisone daily, maximal doses of salmeterol 25 mcg/fluticasone 500 mcg metered dose inhaler, the patient’s ACT score was 15 and pulmonary function tests showed a forced expiratory volume in one second (FEV_1_) lower than 80% and AEC of 406/mm^3^; therefore a clinical diagnosis of severe eosinophilic asthma was made and biological therapy with benralizumab was started. The standard recommended dosage regimen for severe asthma was used, i.e., 30 mg by subcutaneous injection every 4 weeks for the first 3 months, and then every 8 weeks thereafter. Four months after starting treatment with benralizumab, her blood eosinophils were completely depleted. She managed to suspend her daily OCS after the third dose. Eight months later, her respiratory symptoms are well-controlled, with an ACT score of 22, and she has reported no further asthmatic or other systemic exacerbations since starting benralizumab. Her BVAS score is now 0. The patient has reported optimal adherence and no adverse drug reactions to the drug administered.

## Discussion

ANCA-negative EGPA tends to present more frequently in younger patients than its ANCA-positive counterpart (Zwerina et al., 2009) ANCA-negative patients usually present with eosinophilic infiltration in tissues such as the lungs, heart, and gastrointestinal tract; by contrast, ANCA-positive patients tend to present with a vasculitic disease pattern such as glomerulonephritis, pulmonary hemorrhage, and mononeuritis multiplex (Sinico et al., 2005). An ANCA titer is not the perfect discriminatory marker between vasculitic and non-vasculitic EGPA, as ANCA-negative patients sometimes present with true vasculitic features as reported in a French study (Cottin et al., 2016). Our patient developed recurrent pericarditis which is not considered a direct manifestation of vasculitis, but found to be present in 18% of ANCA-negative EGPA patients (Cottin et al., 2017), and had symptoms suggestive of EGPA-related vasculitis in the form of purpura despite no histopathological confirmation of this. Asthma is a cardinal symptom of EGPA present in most patients and is generally late onset. It is classically severe and corticosteroid-dependent (Dávila González et al., 2019). Our case report highlights the importance of identifying patients with early-onset severe asthma who could have an underlying EGPA.

Daily OCS, the mainstay of treatment in EGPA, is associated with many adverse effects. Oral corticosteroid use is associated with osteoporosis, hypertension, obesity, type 2 diabetes, gastrointestinal ulceration and bleeding, fractures, and cataracts (Sullivan et al., 2018). Use of systemic corticosteroids and the risk of developing systemic corticosteroid-related complications in patients with severe asthma follows a statistically significant linear cumulative dose-response (Canonica et al., 2019). Besides the side-effect burden, in our patient, OCS only partially resolved her symptoms.

Eosinophilic inflammation plays a prominent role in EGPA. Targeting eosinophils in EGPA is important as its pathogenesis is IL-5 mediated and not only ANCA -mediated (Furuta et al., 2019) IL-5 is a powerful pro-inflammatory cytokine that is necessary for the maturation, proliferation, activation, and migration of eosinophils (Pelaia et al., 2019). Activated eosinophils exert proinflammatory effects by releasing cytotoxic granule proteins and lipid mediators, thereby inducing tissue damage and inflammation (Furuta et al., 2019). Timely treatment targeting IL-5 can reduce morbidity and hospitalizations. In September 2021 EMA recommended granting an extension of indication to mepolizumab at a dose of 300 mg monthly as an add-on treatment in EGPA (New add-on treatment for rare autoimmune inflammatory disorder | European Medicines Agency). There is an ongoing phase 3 study comparing the efficacy of benralizumab and mepolizumab in EGPA (Efficacy and Safety of Benralizumab in EGPA Compared to Mepolizumab.–Full Text View–ClinicalTrials.gov). An ongoing phase 2 study is assessing the efficacy and safety of benralizumab in EGPA (Benralizumab in the Treatment of Eosinophilic Granulomatosis With Polyangiitis (EGPA) Study–Full Text View–ClinicalTrials.gov).

Benralizumab differs as it is a humanized monoclonal antibody that binds to the IL-5 receptor alpha on eosinophils blocking the activation of IL-5/IL-5R pathway determining a reduction of eosinophil proliferation, maturation, and migration from the bone marrow to target organs (Dávila González et al., 2019). It also lacks a fucose sugar residue in the CH2 region of the Fc domain; this afucosylation enables benralizumab to bind with high affinity to the RIIIa region of the Fcγ receptor found on NK cells, macrophages, and neutrophils, thus strongly inducing antibody-dependent cell-mediated cytotoxicity of eosinophils and basophils. This double function makes benralizumab an intriguing prospect in hypereosinophilic disorders and several case reports and case series have reported encouraging results in its use in the management of EGPA, with improvement of respiratory symptoms (Coppola et al., 2020; Miyata et al., 2021; Martínez-Rivera et al., 2021). Benralizumab has also been reported to improve cutaneous vasculitis (Menzella et al., 2021) and cardiac and brain vasculitis (Kolios et al., 2021) in EGPA. When used in EGPA benralizumab can nullify ANCA titers (Kolios et al., 2021); (Laorden et al., 2021) or decrease them (Menzella et al., 2021; Miyata et al., 2021; Takenaka et al., 2019). Most patients in the aforementioned reports were diagnosed in the 5th decade of life, while one patient was diagnosed in the 3rd decade of life, in contrast with our patient who was diagnosed with EGPA at a much younger age.

Benralizumab has enabled our patient to achieve control of her asthma, stop OCS completely and avoid other organ system exacerbations of EGPA. The outcomes of ongoing clinical trials are necessary to confirm the efficacy and safety of benralizumab treatment in patients affected by EGPA.

## Data Availability

The raw data supporting the conclusion of this article will be made available by the authors, without undue reservation.

## Abbreviations

ACT, asthma control test; AEC, absolute eosinophilic count; ANA, anti neutrophil antibodies; ANCA, antineutrophil cytoplasmic antibodies; CH2, hydrocarbon; CT, computerized tomography scan; EGPA, eosinophilic granulomatosis with polyangiitis; ENA, extractable nuclear antigens; Fc, fragment crystallizable; FDA, food and drug administration; FEV1, forced expiratory volume in 1 second; ICS, inhaled corticosteroids; IgM, immunoglobulin M; IL-5, interleukin 5; IL-5R, interleukin 5 receptor; IU, international units; LABA, long acting beta 2 agonists; MRI, magnetic resonance imaging; NK, natural killer; OCS, oral corticosteroids.
